# Highly pathogenic avian influenza A virus H5N1 NS1 protein induces caspase-dependent apoptosis in human alveolar basal epithelial cells

**DOI:** 10.1186/1743-422X-7-51

**Published:** 2010-03-03

**Authors:** Chuanfu Zhang, Yutao Yang, Xiaowei Zhou, Xuelin Liu, Hongbin Song, Yuxian He, Peitang Huang

**Affiliations:** 1 Institute of Biotechnology, Academy of Military Medical Sciences, Beijing 100071, PR China; 2 Institute of Disease Control and Prevention, Chinese Academy of Military Medical Sciences, Beijing 100071, PR China; 3 Institute of Pathogen Biology, Chinese Academy of Medical Sciences and Peking Union Medical College, Beijing 100730, PR China; 4 Beijing Institute for Neuroscience, Capital Medical University, Beijing, 100069, PR China

## Abstract

**Background:**

It is widely considered that the multifunctional NS1 protein of influenza A viruses contributes significantly disease pathogenesis by modulating a number of virus and host-cell processes, but it is highly controversial whether this non-structural protein is a proapoptotic or antiapoptotic factor in infected cells.

**Results:**

NS1 protein of influenza A/chicken/Jilin/2003 virus, a highly pathogenic H5N1 strain, could induce apoptosis in the carcinomic human alveolar basal epithelial cells (A549) by electron microscopic and flow cytometric analyses. NS1 protein-triggered apoptosis in A549 cells is via caspase-dependent pathway.

**Conclusions:**

Influenza A virus NS1 protein serves as a strong inducer of apoptosis in infected human respiratory epithelial cells and plays a critical role in disease pathogenesis.

## Background

The influenza A virus, which contains eight segmented and negative-stranded RNAs as its genome, is a globally important human and animal respiratory pathogen responsible for both seasonal "flu" outbreaks and periodic world-wide pandemics. In recent years, the highly pathogenic avian influenza A virus H5N1 has been frequently transmitted to human and caused a mortality rate of >30%, raising serious worldwide concern about a severe influenza pandemic. Although considerable efforts, the mechanism accounting for the severity of human H5N1 infection remains elusive. It has been demonstrated that influenza viruses can induce apoptosis in numerous cell types, both in vivo [1-3] and in vitro [4-12]. Recently, the apoptosis was observed among the alveolar epithelial cells of two patients who died of H5N1 infection, suggesting a possible role of apoptosis in H5N1 pathogenesis in humans [13].

Several viral factors, including neuraminidase, M1, NS1, and PB1-F2, from different strains of human influenza viruses could induce or inhibit apoptosis in human cells [7,14-18]. The multifunctional NS1 protein is widely considered as a virulence factor and contributes significantly disease pathogenesis by modulating a number of virus and host-cell processes [19-22]. Prominently, it is hotly debated that whether the NS1 protein is a proapoptotic or antiapoptotic factor in infected cells [23]. For example, the NS1 proteins derived from H5N9 or H5N1 could induce apoptosis in MDCK, HeLa cells or human airway epithelial cells [8,24]; in sharp contrast, the NS1 proteins from H1N1 or H3N2 were reported to down-regulate apoptosis in MDCK and Vero cells [18,25]. Furthermore, while it was shown that the H5N1 NS1 protein was capable of inducing caspase pathway-dependent apoptosis [26], the NS1 from H1N1 could activate PI3K/Akt pathway to mediate antiapoptotic signaling responses [27]. It is possible that these diverse observations might be resulted from the differences of virus subtypes and strains, as well as the host cell system being used, highlighting that further characterization of the NS1 protein and its mechanism involved in the induction of apoptosis is highly essential for understanding the pathogenesis of influenza A viruses. In this study, we demonstrated that the expression of NS1 proteins of influenza A/chicken/Jilin/2003(H5N1)could induce apoptosis in the carcinomic human alveolar basal epithelial cells (A549) via the caspase-dependent pathway, providing further evidences to support that the H5N1 NS1 plays a critical in disease pathogenesis.

## Materials and methods

### Viruses and cells

Influenza A/chicken/Jilin/2003(H5N1)virus was grown in the allantoic cavities of 10-day-old embryonated chicken eggs. A549 cells were passaged in Dulbecco's modified Eagle's tissue culture medium (DMEM) containing 10% fetal calf serum at 37°C in a 5% CO_2 _incubator. For immunoblot analysis, confluent cell monolayers grown in 25-mm dishes were lysed in immunoprecipitation assay buffer containing 150 mM NaCl, 1.0% Nonidet P-40 (NP-40), 0.5% deoxycholate, 0.1% sodium dodecyl sulfate (SDS), and 50 mM Tris-HCl (pH 8.0). Lysates were clarified by centrifugation for 10 min at 13,000 *g *and supernatants were used for immunoblot analysis.

### Construction of NS1-expressing plasmid

Total RNA was extracted from the cell lysates using the QIAamp viral RNA mini kit (Qiagen, Hilden, Germany). The full-length NS1 gene from influenza A/chicken/Jilin/2003 was amplified using the SuperScript III one-step reverse transcription-PCR (RT-PCR) system with Platinum *Taq *high-fidelity polymerase (Invitrogen, Carlsbad, CA). The construction of plasmid pCMV-myc/NS1 followed standard cloning procedures. Competent *Escherichia coli *DH5α cells were transformed with the plasmids, and the plasmids were amplified and purified using a high-purity plasmid purification kit (Qiagen).

### Electron microscopic analysis

A549 cells were transfected with pCMV-myc/NS1, using Lipofectamine 2000 reagent (Invitrogen). After 24 h, cells were collected, digested, washed with phosphate buffered solution (PBS), fixed with 4% glutaraldehyde for 2 h, and then fixed with osmium tetroxide for 1 h, stained with uranium acetate, embedded into 6.8^# ^epoxide resin. After sectioning into ultra-thin slices, the cells were stained with lead citrate and examined under transmission electron microscopy.

### Flow cytometric analysis

To determine the apoptosis rate, an Annexin V-FITC apoptosis detection kit (BD Pharmingen, San Diego, CA) was used to detect early apoptotic activity according to the manufacturer's instructions, with slight modifications. After 24 h transfected as described above, the A549 cells were harvested and washed twice with ice-cold PBS and resuspended in 100 ml of binding buffer. A total of 5 ml of Annexin V-FITC and 10 ml of propidium iodide (PI) were added and the mixture was incubated for 30 min in the dark. Finally, 400 ml of binding buffer was added to the cells, the mixture were analyzed with a Flow cytometer (Becton Dickinson Co., San Jose, CA), using an FITC signal detector (FL1) for Annexin V staining and a phycoerythrin emission signal detector for PI staining. The apoptotic percentage of 10,000 cells was determined. All the experiments reported in this study were performed three times. The data were analyzed using WinMDI 2.8 software (Scripps Institute, La Jolla, CA) for calculation of percentage cells with apoptosis per group.

### Expression of caspase-9 and caspase-3

The expression of caspase-9 and caspase-3 in NS1-transfected A549 cells were measured by Western-blot analysis. Briefly, monolayer of cells transfected with pCMV-myc/NS1 was lysed with ice-cold lysis buffer (150 mM Tris-HCl, pH 8.0, 50 mM NaCl, 1 mM EDTA, 0.5% Nonidet P-40, 1 tablet Complete Mini protein inhibitor mixture/10 ml (Roche Applied Science) and 0.7 μg/ml pepstatin), and the lysates were clarified by centrifugation at 20,000 *g *for 10 min at 4°C. Caspase-9 and caspase-3 activities were determined according to the supplemental protocols of the Caspase-9/Mch6 Colorimetric Assay kit and Caspase-3/CPP32 Colorimetric Protease Assay kit (MBL, Nagoya, Japan), respectively. Substrate cleavage, which resulted in the release of pNA (405 nm), was measured using a Multiskan Ascent plate reader.

## Results

### Influenza A virus H5N1 NS1 protein induced apoptosis in A549 cells

**Figure 1:**
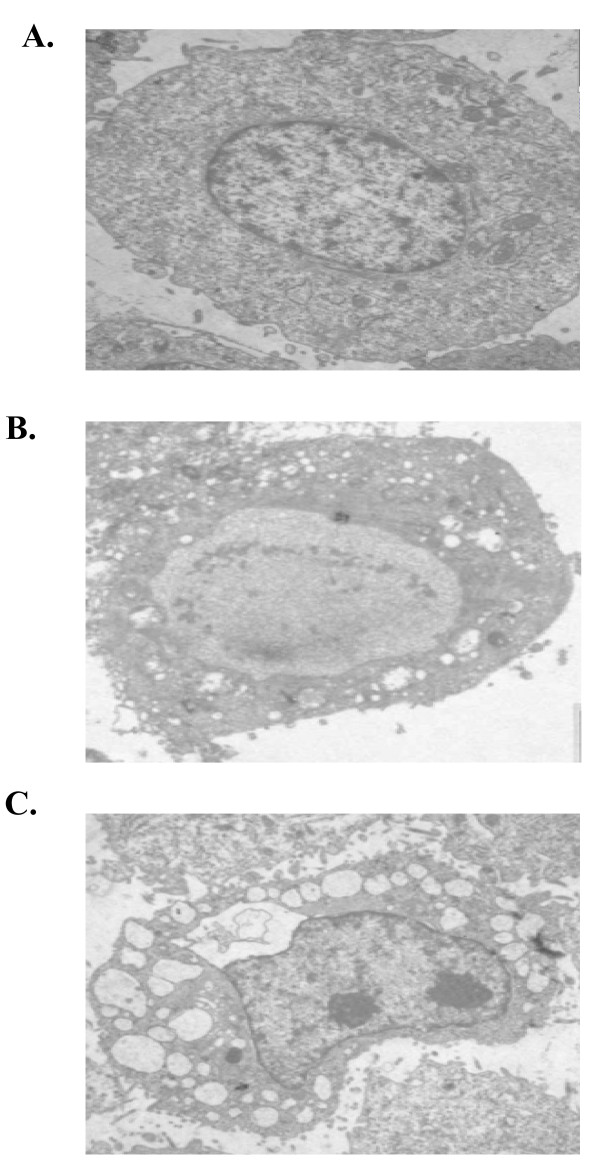
**H5N1 NS1 protein induces apoptosis in A549 cells visualized by transmission electron microscopy**. Normal cell (A) and transfected cell with pCMV-Myc empty vector (B) show that the nuclear shapes are intact. H5N1 NS1-transfected cell shows chromatins condensed, shrunk and aggregated along inside the nuclear membrane, and reveals the apoptotic bodies. (× 5,000).

The biological activities of influenza A virus NS1 proteins are likely to be strain- and/or cell-type specific [28]. Here, we studied whether the NS1 of influenza A/chicken/Jilin/2003(H5N1)virus could induce apoptosis in A549 cells. The NS1 gene of this strain was amplified by RT-PCR and cloned into a mammalian expression vector to construct pCMV-myc/NS1. The expression of NS1 protein in A549 cells was assessed after transfection with the plasmid by Western blot analysis. We examined the morphological changes and ultrastructural features of the A549 cells transfected with the NS1-expressing vectors under a transmission electron microscopy. As shown in Fig. 1C, the NS1-transfected cells appeared characteristics of apoptotic cells, including nuclear condensation and chromatin aggregation to the nuclear membrane. Typically, the apoptotic bodies were found in some cells. In the contrast, the cells transfected with the empty vector (Fig. 1B) and normal cells (Fig. 1A) revealed normal silhouettes.

**Figure 2:**
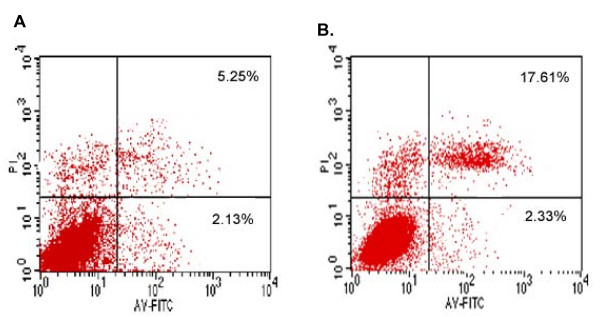
**Flow cytometric analysis of H5N1 NS-induced apoptosis**. The dot plot diagrams represent typical apoptotic and necrotic cell populations detected by Annexin V-FITC and PI staining. A. A549 cells transfected with the empty PCMV-Myc vectors. B. A549 cells transfected with pCMV-Myc/NS1. The lower left quadrants of the panels show viable intact cells, which were negative for Annexin V-FITC binding and excluded PI staining (FITC^-^/PI^-^); the upper right quadrants show nonviable, necrotic cells, which were positive for Annexin V-FITC binding and PI uptake (FITC^+^/PI^+^). The lower right quadrants represent apoptotic cells, positive for Annexin V-FITC and negative for PI (FITC^+^/PI^-^).

Further, the A549 cells expressing NS1 protein were stained with annexin V-FITC and PI and analyzed by flow cytometry. As showed in Fig. 2B, H5N1 NS1-transfected cells were 2.33% Annexin V^+^/PI^- ^(early apoptosis) and 17.61% Annexin V^+^/PI^+ ^(latter apoptosis), while the empty vector-transfected cells were 2.13% Annexin V^+^/PI^- ^and 5.25% Annexin V^+^/PI^+ ^(Fig. 2A). Collectively, these results suggested that the NS1 protein of influenza A virus H5N1 was able to induce apoptosis in A549 cells.

### Involvement of caspases in NS1-induced apoptosis

**Figure 3:**
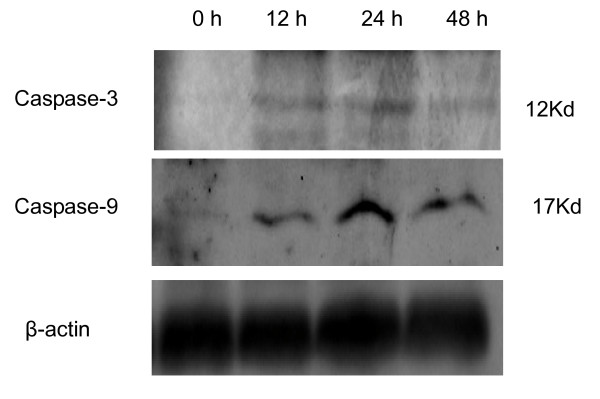
**Caspase-3 and caspase-9 activation in NS1-transfected A549 cells**. The cells were transfected with the plasmid pCMV-Myc/NS1 for 0 h, 12 h, 24 h, and 48 h. Intracellular caspase-3 and caspase-9 activation were detected by Western blotting. The β-actin was used as a loading control.

**Figure 4:**
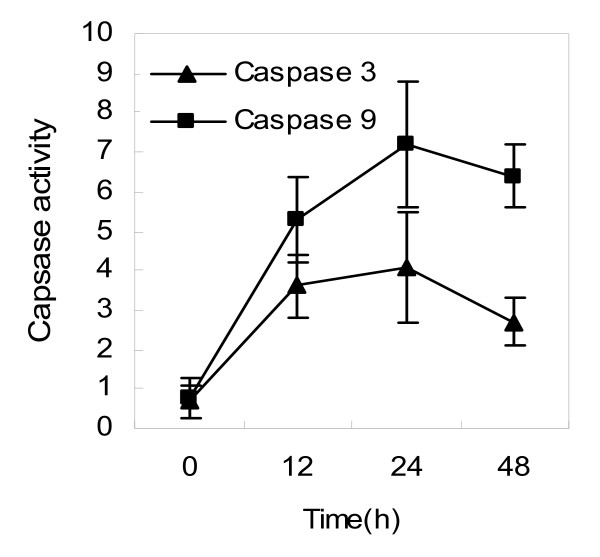
**Enzyme activities of caspase-3 and caspase-9 in NS1-transfected A549 cells**. Enzyme activities were detected by Caspase-3/CPP32 Colorimetric Protease Assay and Caspase-9/Mch6 Colorimetric Assay, respectively.

Previous studies suggested that avian influenza virus A/HK/483/97 (H5N1) NS1 protein-induced apoptosis in a human airway epithelial cells, NCI-H292, was caspase pathway-dependent [26]. Here, we performed experiments to investigate whether the caspase pathways were involved in the apoptosis induced by NS1 proteins derived from the influenza A/chicken/Jilin/2003 virus. To this end, A549 cells were transfected with the NS1-expressing plasmid and the cell lysate was prepared as described in Materials and Methods. First, the expression of caspase-9 and caspase-3 were detected by Western-blotting analysis and found that the apoptosis-related caspase-9 and caspase-3 were activated in H5N1 NS1-transfected A549 cells (Fig. 3). The active fragments of caspase-9 and caspase-3 could be detected at 12 h, 24 h, 48 h post-transfection. Second, we measured the enzyme activities of caspase-9 and caspase-3 and the results revealed that both apoptosis-associated enzymes were activated in the NS1-transfected A549 cells (Fig. 4). Therefore, our data verified that the NS1 protein of influenza A virus H5N1 can induce caspase-dependent apoptosis in A549 cells.

## Discussion

A number of studies have demonstrated that influenza virus infection can induce apoptosis in a variety of cell lines, but the mechanism of this effect remains to be characterized [1-12]. Previous studies thought that apoptosis is a host defense response that limits the virus replication [29], but recent evidence showed that the induction of apoptosis is essential for virus mRNA synthesis and propagation [16,30,31]. Viral proteins from different strains of human influenza viruses have been reported to have proapoptotic or antiapoptotic functions in human cells [7,14-18]. The importance of non-structural protein NS1 in the viral pathogenesis, especially its role in the virus-induced apoptosis, has been recently underlined [8,17,23,25,27]. A highly controversial question is whether this multifunctional protein is a proapoptotic or antiapoptotic factor in infected cells. Schultz-Cherry *et al*. reported that the expression of NS1 protein of H5N9 was sufficient to induce apoptosis in MDCK and Hela cells [8]; Lam *et al *demonstrated recently that H5N1 NS1 protein could induce apoptosis in human airway epithelial cells (NCI-H292) [26]. It was also reported that a poor-apoptosis-inducer strain could be converted into a strong-inducer strain by the NS1 gene substitution by reverse genetics, and vice versa [25]. Discordantly, the NS1 protein has been also shown to inhibit apoptosis. For examples, Zhirnov *et al*. found that H1N1 NS1 protein had IFN-dependent antiapoptotic potential and down-regulated the apoptotic response in virus-infected in cultured cells and chicken embryos [18]; Ehrhardt *et al *found that the NS1 proteins of H1N1 and H7N7 activated the phosphatidylinositol 3-kinase (PI3K/Akt) pathway to mediate antiapoptotic signaling responses [27]. The discrepancy has confused our understanding to the role of NS1 protein in influenza virus-induced apoptosis, highlighting that further characterization is needed to exclude the possibility resulted from the different cell lines and virus strains in each experiment [20]. In the present study, we cloned the NS1 gene from the influenza A/chicken/Jilin/2003 virus (H5N1) and expressed the NS1 protein in the human alveolar basal epithelial cell A549. With electron microscopic and flow cytometric analyses we verified that the expression of H5N1 NS1 protein was capable to induce apoptotic events posttransfection in the A549 cells. The biological basis accounting for the severity of H5N1 infection in humans is still unknown. Our current experimental data, together with the apoptotic observations in the alveolar epithelial cells of two patients who died of H5N1 infection [13], have provided convincing evidence to the critical role of H5N1-encoded NS1 protein in inducing apoptosis.

Apoptosis, or programmed cell death, involves a series of biochemical events that lead the cells undergo characteristic morphological changes, including blebbing, shrinkage, nuclear fragmentation, chromatin condensation, and chromosomal DNA fragmentation [32]. Sequential activation of caspases cascade plays a central role in the execution-phase of cell apoptosis. Recently, It has been reported that avian influenza virus A/HK/483/97(H5N1) NS1 protein-induced apoptosis in human lung epithelial cells is mainly via the caspase-dependent pathway [26], which encourages further investigation into the potential of the NS1 as a novel therapeutic target. To delineate the apoptotic pathway, we measured the expression of caspase-3 and caspase-9 by Western blotting and their enzyme activities by colorimetric Assay. The data demonstrated that these two apoptosis markers were significantly activated in H5N1 NS1-transfected A549 cells, consistent with the previous studies. Therefore, we conclude that the NS1 protein encoded by avian influenza A virus H5N1 can induce apoptosis in human respiratory epithelial cells via the caspase-dependent pathway. Since the apoptotic destruction of host cells has been thought to contribute the severe disease, we can predict that drugs that can prevent this specific process may reduce disease severity and improve clinical outcomes. Therefore, further investigations to clarify whether the NS1 protein and its apoptotic pathway is worthwhile therapeutic targets for treating H5N1 infection in humans should be considered.

## Competing interests

The authors declare that they have no competing interests.

## Authors' contributions

CFZ, YTY and XWZ mainly carried out gene cloning, western blot, Flow cytometric analysis, and wrote the manuscript. XLL contributed to Electron microscopic analysis. HBS, YXH and PTH conceived the studies and participated in experimental design and coordination. All authors read and approved the final manuscript.
